# G2P[4]-RotaTeq Reassortant Rotavirus in Vaccinated Child, United States

**DOI:** 10.3201/eid2111.150850

**Published:** 2015-11

**Authors:** Sunando Roy, Kunchala Rungsrisuriyachai, Mathew D. Esona, Julie A. Boom, Leila C. Sahni, Marcia A. Rench, Carol J. Baker, Mary E. Wikswo, Daniel C. Payne, Umesh D. Parashar, Michael D. Bowen

**Affiliations:** Centers for Disease Control and Prevention, Atlanta, Georgia USA (S. Roy, K. Rungsrisuriyachai, M.D. Esona, M.E. Wikswo, D.C. Payne, U.D. Parashar, M.D. Bowen);; Texas Children’s Hospital, Houston, Texas USA (J.A. Boom, L.C. Sahni, C.J. Baker);; Baylor College of Medicine, Houston (M.A. Rench, C.J. Baker)

**Keywords:** rotavirus, RotaTeq, NSP2 gene, nonstructural protein 2 gene, reassortant, rotavirus, viruses

**To the Editor:** Group A rotaviruses (RVAs) are a leading cause of acute gastroenteritis-associated deaths among children <5 years of age in developing countries (*1*). The genome of RVA consists of 11 double-stranded RNA segments that code for 11 or 12 viral proteins (VP1–VP4, VP6, VP7, nonstructural protein 1 [NSP1]–NSP5/6) (*2*). In 2008, the Rotavirus Classification Working Group established a system of extended classification that was based on the sequences of all 11 gene segments and used the notations Gx-P[x]-Ix-Rx-Cx-Mx-Ax-Nx-Tx-Ex-Hx for the genes VP7, VP4, VP6, VP1–VP3, NSP1–NSP5, respectively (*3*). Similar to other RNA viruses, RVAs show high genomic diversity, which is generated primarily through point mutations, reassortment, rearrangement, and recombination events.

In 2006 and 2008, two live-attenuated vaccines, RotaTeq (Merck, Whitehouse Station, NJ, USA) and Rotarix (GlaxoSmithKline, Rixensart, Belgium), respectively, were introduced in the United States (*4*). RotaTeq is a pentavalent human bovine reassortant vaccine that contains 4 G types (G1, G2, G3, and G4; VP7 gene) plus the P[8] VP4 type on a bovine WC3 (G6P[5]) backbone (*5*). In 2012, Bucardo et al. reported finding a vaccine-derived nonstructural protein 2 (NSP2) gene in 2 wild-type RVA strains with a G1P[8] genogroup 1 backbone (*6*). Each of these strains had been found during routine surveillance in Nicaragua, where RotaTeq was introduced in 2006, suggesting reassortment of the vaccine strain with circulating wild-type strains. The authors also examined alignments of the NSP2 gene and found no differences at functional domains between the vaccine-derived NSP2 and the circulating wild-type NSP2 (*6*). This finding could explain why a vaccine-derived NSP2 reassortant was viable.

During the 2010–11 surveillance season, the New Vaccine Surveillance Network identified an RVA strain, RVA/human-wt/USA/2011729115/2011/G2P[4] (2011729115), which also contained a vaccine-derived NSP2 gene. This specimen was obtained from a 4-year-old child through routine active surveillance in the emergency department at Texas Children’s Hospital (Houston, TX, USA). RVA double-stranded RNA was extracted from a fecal sample from the child by using Trizol reagent (Life Technologies, Grand Island, NY, USA). The sequencing templates were prepared by using sequence-independent whole-genome reverse transcription PCR amplification (*7*) with slight modifications. PCR amplicons were sequenced by the Illumina Miseq 150 paired-end method at the Genomics Laboratory, Hudson Alpha Institute for Biotechnology (Huntsville, AL, USA). Illumina sequence reads were analyzed by using CLC Genomics Workbench 6.0. (http://www.clcbio.com/products/clc-genomics-workbench/). A combination of de novo assembly followed by mapping to a G2P[4] reference strain was used to obtain the full-length genome of strain 2011729115. The sequences were submitted to GenBank under accession nos. KR701624–KR701634. Genotype assignment for each gene was performed by using a combination of BLAST (http://blast.ncbi.nlm.nih.gov/Blast.cgi) and RotaC 2.0 (http://rotac.regatools.be/). For each gene, multiple alignments were made by using the MUSCLE algorithm implemented in MEGA 5.1 (*8*). Maximum-likelihood trees were constructed for each gene in PhyML 3.0 (http://www.atgc-montpellier.fr/phyml/) by using the optimal model for each alignment as identified by jModeltest 2 (TrN+I) and approximate-likelihood ratio test (aLRT) statistics computed for branch support (*9*,*10*).

The full genotype constellation for strain 2011729115 is G2-P[4]-I2-R2-C2-M2-A2-N2-T2-E2-H2, which is a DS-1 like constellation. The G2P[4] strains from the 2010–11 season shared 97.6%–100% nt identity for the NSP2 gene, except for strain 2011729115, which exhibited only 86.8%–87.3% nt identity with the other circulating G2P[4] strains. BLAST analysis of sequences deposited in GenBank indicated that the 2011729115 NSP2 gene was 100% identical to published RotaTeq NSP2 gene sequences and clustered with the 5 RotaTeq NSP2 sequences in phylogenetic analysis (Figure). The NSP2 protein functions as a single-stranded RNA binding protein, nucleic acid helix destabilizer, nucleoside triphosphatase (NTP), nucleoside diphosphate kinase (NDP), and RNA triphosphatase (RTPase) (*2*). No amino acid differences in the RNA binding and NDP/NTP/RTPase domains were found between the NSP2 gene from strain 2011729115 and the G2P[4] strains (data not shown), suggesting that this vaccine-derived NSP2 protein can function like the wild-type version. 

**Figure:**
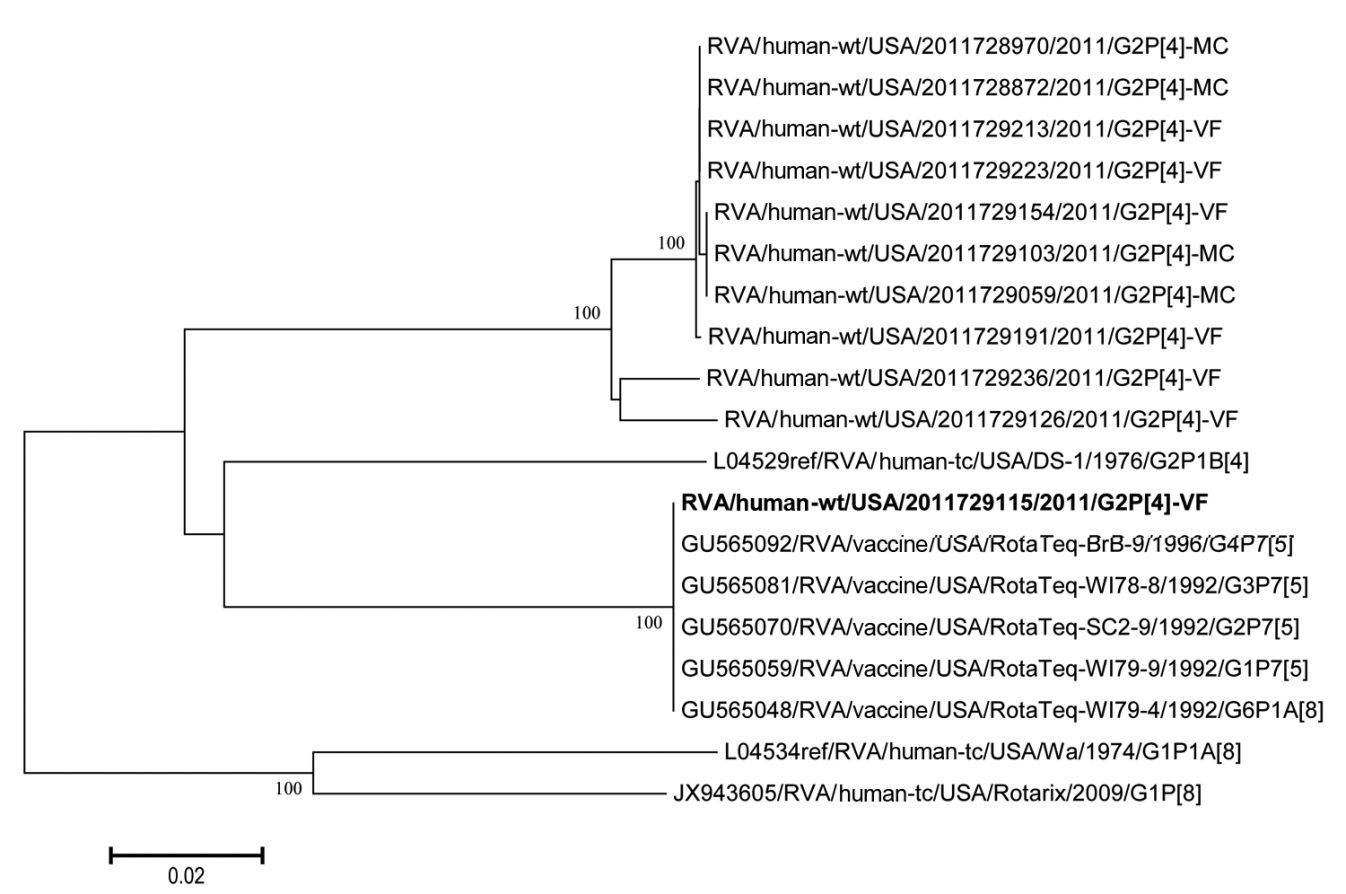
Maximum-likelihood tree for the rotavirus (RVA) nonstructural protein 2 (NSP2) gene showing phylogenetic clustering with wild-type G2P[4] strains identified during the 2010–11 season, United States, and RotaTeq (Merck, Whitehouse Station, NJ, USA) vaccine strains. The tree was created by using MEGA 5.1 (*8*). Approximate-likelihood ratio test values >70% are shown next to supported nodes. Boldface indicates strain 2011729115. Scale bar indicates number of nucleotide substitutions per site.

In conclusion, we identified an NSP2 gene RotaTeq reassortant in an RVA with a DS-1 like genotype. Although the child from whom the NSP2 reassortment specimen was obtained had been vaccinated with a complete regimen of 3 doses of RotaTeq in 2007, no other RotaTeq genes could be detected in the fecal sample. This finding could suggest a reassortment event and infection independent of the vaccination in 2007, although precisely where this reassortment event took place is difficult to ascertain. The existence of a viable NSP2 wild-type reassortant was also proposed by Bucardo et al. (*6*). Whether the RotaTeq NSP2 gene provides any fitness advantage to the virus during infection of vaccinated children also is unclear; during the 2010–11 season, other cases of G2P[4]-associated gastroenteritis in vaccinated children, in which the RotaTeq NSP2 gene was lacking, were reported. Our finding of strain RVA/human-wt/USA/2011729115/2011/G2P[4], a wild-type RVA with a vaccine-derived NSP2 gene, in a child with acute gastroenteritis in the United States highlights the need for continued rotavirus surveillance, domestically and internationally, after vaccine introduction.
